# Supplementary material for: mRNA vaccine-induced T cells respond identically to SARS-CoV-2 variants of concern but differ in longevity and homing properties depending on prior infection status
Source: eLife. 2021 Oct 12;10:e72619. doi: 10.7554/eLife.72619 (PMC8545397; doi:10.7554/eLife.72619)
Supplement: Transparent reporting form [file elife-72619-transrepform1.docx]

***eLife’s* transparent reporting form**

We encourage authors to provide detailed information *within their submission* to facilitate the interpretation and replication of experiments. Authors can upload supporting documentation to indicate the use of appropriate reporting guidelines for health-related research (see [EQUATOR Network](http://www.equator-network.org/%20)), life science research (see the [BioSharing Information Resource](https://biosharing.org/)), or the [ARRIVE guidelines](http://www.plosbiology.org/article/info:doi/10.1371/journal.pbio.1000412) for reporting work involving animal research. Where applicable, authors should refer to any relevant reporting standards documents in this form.

If you have any questions, please consult our Journal Policies and/or contact us: [editorial@elifesciences.org](mailto:editorial@elifesciences.org).

**Sample-size estimation**

- You should state whether an appropriate sample size was computed when the study was being designed
- You should state the statistical method of sample size computation and any required assumptions
- If no explicit power analysis was used, you should describe how you decided what sample (replicate) size (number) to use

Please outline where this information can be found within the submission (e.g., sections or figure legends), or explain why this information doesn’t apply to your submission:

A sample size calculation was not performed when the study was being designed, because these were COVID-19 patient specimens that were tested as they became available and we based the number of samples we analyzed on 1) the numbers of eligible patients for which we could do 3 large blood draws at matched longitudinal timepoints (which ended up being 11 patients x 3 timepoints = 33 longitudinal patient specimens), and 2) the numbers of total samples we could analyze by high-dimensional CyTOF to characterize various types of SARS-CoV-2-specific T cells (5 test conditions for each patient specimen, totaling 165 high-dimensional datasets from the 33 specimens). In our study, we always indicated the statistical significance of our results, and only drew conclusions based on statistically significant results.

**Replicates**

- You should report how often each experiment was performed
- You should include a definition of biological versus technical replication
- The data obtained should be provided and sufficient information should be provided to indicate the number of independent biological and/or technical replicates
- If you encountered any outliers, you should describe how these were handled
- Criteria for exclusion/inclusion of data should be clearly stated
- High-throughput sequence data should be uploaded before submission, with a private link for reviewers provided (these are available from both GEO and ArrayExpress)

Please outline where this information can be found within the submission (e.g., sections or figure legends), or explain why this information doesn’t apply to your submission:

All replicates were biological not technical, and the figure legends and Methods describes the statistical tests used. There was one outlier specimen which was included in all the analysis except in the one figure panel where an additional analysis was conducted whilst excluding this outlier, with the justification for this additional analysis detailed in the Results section. The exclusion of the donor in this one panel is clearly indicated in the figure and figure legend.

**Statistical reporting**

- Statistical analysis methods should be described and justified
- Raw data should be presented in figures whenever informative to do so (typically when N per group is less than 10)
- For each experiment, you should identify the statistical tests used, exact values of N, definitions of center, methods of multiple test correction, and dispersion and precision measures (e.g., mean, median, SD, SEM, confidence intervals; and, for the major substantive results, a measure of effect size (e.g., Pearson's r, Cohen's d)
- Report exact p-values wherever possible alongside the summary statistics and 95% confidence intervals. These should be reported for all key questions and not only when the p-value is less than 0.05.

Please outline where this information can be found within the submission (e.g., sections or figure legends), or explain why this information doesn’t apply to your submission:

The Figure Legends and Methods describes and justifies the statistical tests used. We always show raw (and not normalized) data. The methods of multiple tests used are indicated. We have indicated dispersion and precision measures in the figure legends.

(For large datasets, or papers with a very large number of statistical tests, you may upload a single table file with tests, Ns, etc., with reference to sections in the manuscript.)

**Group allocation**

- Indicate how samples were allocated into experimental groups (in the case of clinical studies, please specify allocation to treatment method); if randomization was used, please also state if restricted randomization was applied
- Indicate if masking was used during group allocation, data collection and/or data analysis

Please outline where this information can be found within the submission (e.g., sections or figure legends), or explain why this information doesn’t apply to your submission:

We had two patient groups: infection-naïve and convalescents. A total of 15 patient specimens (from 5 patients) were assigned to the infection-naïve group, and a total of 18 patient specimens (from 6 patients) were assigned to the convalescent group.

**Additional data files (“source data”)**

- We encourage you to upload relevant additional data files, such as numerical data that are represented as a graph in a figure, or as a summary table
- Where provided, these should be in the most useful format, and they can be uploaded as “Source data” files linked to a main figure or table
- Include model definition files including the full list of parameters used
- Include code used for data analysis (e.g., R, MatLab)
- Avoid stating that data files are “available upon request”

Please indicate the figures or tables for which source data files have been provided:

We have made available the original raw CyTOF datasets through the following Dryad link: https://doi.org/10.7272/Q60R9MMK
